# Rad18 is required for functional interactions between FANCD2, BRCA2, and Rad51 to repair DNA topoisomerase 1-poisons induced lesions and promote fork recovery

**DOI:** 10.18632/oncotarget.7247

**Published:** 2016-02-08

**Authors:** Kaushlendra Tripathi, Chinnadurai Mani, David W Clark, Komaraiah Palle

**Affiliations:** ^1^ Department of Oncologic Sciences, Mitchell Cancer Institute, University of South Alabama, Mobile, Alabama, 36604, USA

**Keywords:** Rad18, FANCD2, Rad51, BRCA2, DNA topoisomerase 1

## Abstract

Camptothecin (CPT) and its analogues are chemotherapeutic agents that covalently and reversibly link DNA Topoisomerase I to its nicked DNA intermediate eliciting the formation of DNA double strand breaks (DSB) during replication. The repair of these DSB involves multiple DNA damage response and repair proteins. Here we demonstrate that CPT-induced DNA damage promotes functional interactions between BRCA2, FANCD2, Rad18, and Rad51 to repair the replication-associated DSB through homologous recombination (HR). Loss of any of these proteins leads to equal disruption of HR repair, causes chromosomal aberrations and sensitizes cells to CPT. Rad18 appears to function upstream in this repair pathway as its downregulation prevents activation of FANCD2, diminishes BRCA2 and Rad51 protein levels, formation of nuclear foci of all three proteins and recovery of stalled or collapsed replication forks in response to CPT. Taken together this work further elucidates the complex interplay of DNA repair proteins in the repair of replication-associated DSB.

## INTRODUCTION

DNA topoisomerase 1 (Top1) is an essential enzyme in higher eukaryotes, which resolves topological barriers during most critical cellular processes involving DNA, including replication, transcription, recombination and repair [1–4]. Top1 modulates DNA topology by introducing a transient single strand break through active site tyrosine (Tyr723) forming a covalent 3′-phosphotyrosyl bond with DNA, which is called Top1-DNA covalent or cleavage complex (Top1cc) [5–8]. The formation of Top1cc is rapidly followed by a second transesterification reaction to reseal the broken strand and to maintain DNA integrity. Topological strain in the DNA drives the broken strand to rotate around the intact strand thereby relieving the extent of supercoiling during the period between the cleavage and religation, allowing transcription or replication to proceed [9]. In normal conditions, the covalently bound Top1cc is transient and mostly undetectable because religation is much faster than cleavage in Top1 catalysis [3, 4]. However, anticancer agents such as camptothecin (CPT) and its clinical analogues (ex. topotecan and irinotecan) intercalate into the Top1 generated DNA nick and inhibit the religation of scissile strand, which greatly prolongs the half-life of Top1cc [9, 10]. These CPT stabilized Top1cc are not known to be cytotoxic by themselves and are readily reversible after removal of the drug. However, prolonged stabilization of Top1cc can create multiple problems. Firstly, failure to relieve supercoiling generated by such processes as transcription and replication can lead to replication stress by creating torsional strain within the DNA [9, 11, 12]. Furthermore, collision between an active replication fork and the Top1cc is capable of generating DNA double strand breaks (DSB) which can introduce mutations or lead to cell death [13–15]. Anticancer agents, such as DNA Top1 poisons as well as others that result in stalled replication forks (hydroxyurea, gemcitabine, and others) are effective as they lead to high levels of DSB in rapidly replicating cancer cells as opposed to quiescent terminally differentiated normal cells [14, 16, 17]. Studies in yeast and human cancer cells identified homologous recombination (HR) as the predominant DNA repair pathway involved in repairing CPT-induced DSB [18–23]. Correspondingly, deficiencies in proteins involved in HR (ex. Rad51) as well as associated proteins such as Rad18, members of the Fanconi anemia (FA) family and the breast cancer associated (BRCA1, BRCA2) proteins sensitize cells to CPT [24–28].

Rad18 belongs to zinc and RING finger family of E3 ubiquitin ligases and its functions are well studied in post-replication repair pathway also known as translesion synthesis (TLS) [29–33]. Rad18 regulates TLS by monoubiquitinating PCNA, thereby triggering a polymerase switch allowing bypass of the bulky adduct-type of DNA lesion [31, 32]. Thus cells defective in Rad18 fail to faithfully replicate DNA over a variety of mutagenic adducts and exhibit hypersensitivity to the presence of these lesions [32]. Moreover, Rad18 has also been to shown to play an important role in repair of DSB by directly binding to Rad51C, a paralog of Rad51 and localize it to sites of DSB to promote HR [26]. We and others have previously shown that Rad18 regulates the FA pathway in response to fork stalling lesions induced by several agents, including CPT [12, 34–37]. These studies demonstrated that Rad18 E3 ligase activity is important for efficient FANCD2 monoubiquitination, as well as its CPT-induced nuclear foci formation and cell survival by timely repair of replication-coupled DSB [12, 37, 38]. The FA proteins (FANCs) associate with replication forks and have been implicated in repair of fork stalling lesions by HR in association with BRCA proteins [39–44]. BRCA2 is also known as FANCD1 and is a member of both families of proteins [40], and recently BRCA1 was given the alternate name FANCS as mutations in BRCA1 are capable of causing FA [41]. An epistatic relationship between FA and BRCA genes has been suggested in repair of replication fork stalling lesions by HR; however, regulation of FA-BRCA proteins and their molecular interactions are rather complex and not well known [42–44]. In this study we focused on the role of Rad18-mediated activation of FANCD2 and its functional relationship with BRCA2 and Rad51 proteins in repair of CPT-induced lesions. Our data show that downregulation of Rad18 or FANCD2 leads to decreased BRCA2 and Rad51 foci formation and co-localization in response to CPT. Consistently, either single gene downregulation or co-depletion with Rad18 resulted in similar levels of sensitivity to CPT and increase in gross chromosomal aberrations (CA). Moreover, Rad18, FANCD2, BRCA2 and Rad51 co-immunoprecipitated and depletion of Rad18 suppressed this interaction, suggesting these proteins work in a common repair pathway to promote accurate repair of CPT-induced replication-coupled DSB.

## RESULTS

### Rad18 is required for proper FANCD2, BRCA2, and Rad51 foci formation in response to CPT-induced DSB

Stabilization of Top1cc by CPT and its clinical analogues induces replication stress and replication-coupled DSB. We recently reported that E3 ligase activity of Rad18 is important for FA pathway activation to efficiently repair CPT-induced DSB and for cell survival [12]. Rad18 has also been shown to interact with Rad51C and promote its recruitment to sites of DSB in an E3 ligase-independent manner [26]. However, several studies established functional interactions and epistatic relationship between FANCD2, BRCA2 and Rad51 in stabilization of stalled replication forks and repair of collapsed forks by HR [41, 44–46]. Rad18 is important for efficient activation of the FA pathway; however, the functional interplay between Rad18 status and FANCD2, BRCA2 and Rad51 proteins in repair of replication-associated DNA lesions is not known. To examine this, we transiently downregulated these genes and examined their influence on each other in response to Top1 poison CPT.

**Figure 1:**
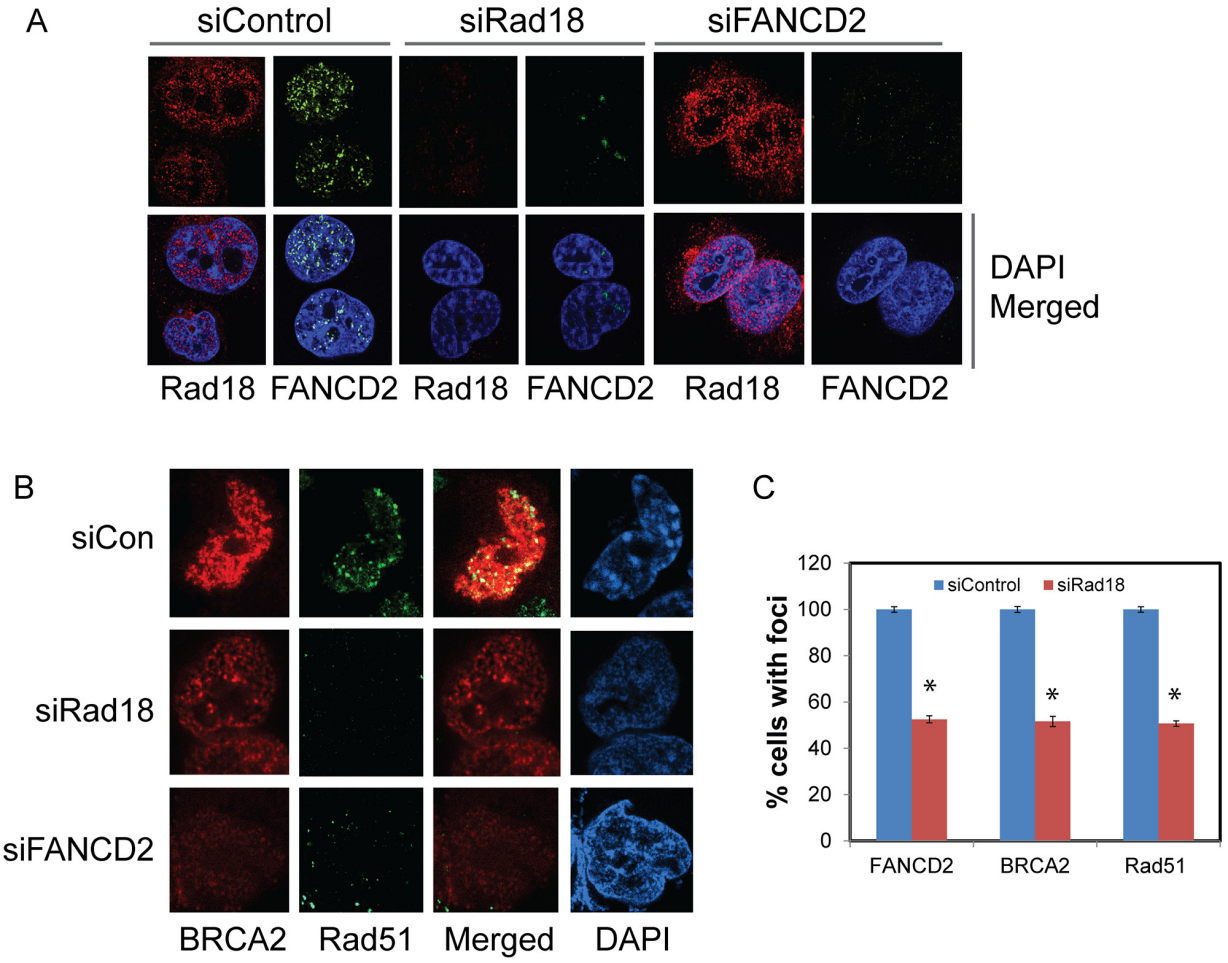
Effect of Rad18 or FANCD2 depletion on Rad18, FANCD2, BRCA2 and Rad51 foci formation in response to CPT H1299 cells were transfected with siRNA directed against either Rad18 or FANCD2 or a control siRNAs and were exposed to 500 nM CPT for 2 hours, fixed and labeled with the indicated antibodies and stained with fluorophore-labeled secondary antibodies and nuclei were stained with DAPI. The immunofluorescence images were acquired using Nikon Ti eclipse confocal microscope at 100X. Cells treated with DMSO did not demonstrate any significant, detectable foci (data not shown). **A.** The effects of knocking down of Rad18 on foci formation of FANCD2, and Rad18 foci formation in FANCD2-knockdown cells in response to CPT. **B.** The effects of depleting either Rad18 or FANCD2 on foci formation of BRCA2 and Rad51 in response to CPT. The merged panel shows the extent of co-localization between BRCA2 and Rad51 under these conditions. **C.** Graph showing the normalized mean of three independent experiments with bars representing ± S.D. * Denotes statistical significance compared to their respective controls (P<0.05).

**Figure 2:**
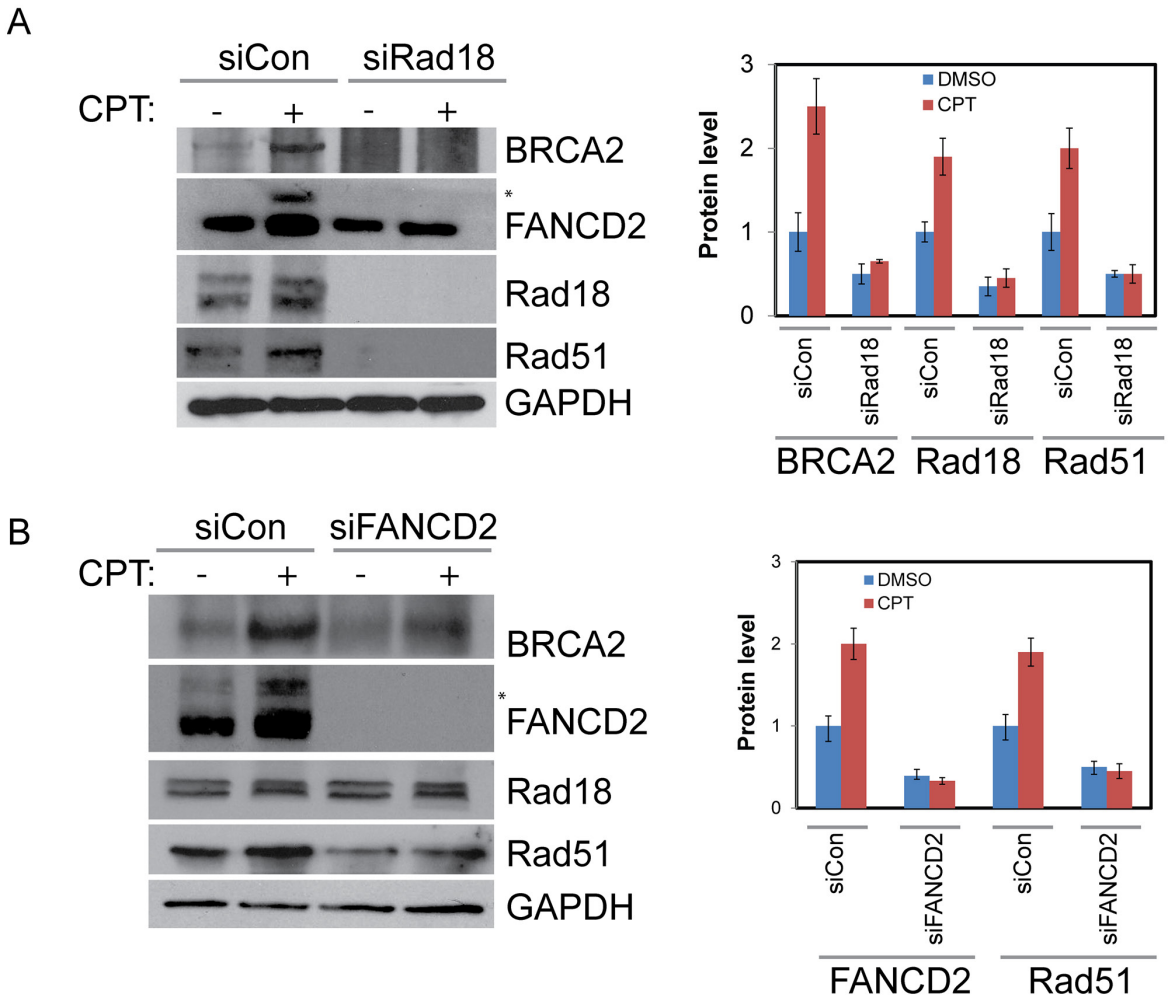
Rad18 is important for proper activation of FANCD2 and both Rad18 and FANCD2 are important for the stability of Rad51 and BRCA2 H1299 cells were depleted of Rad18 or FANCD2 by siRNAs or treated with control siRNAs for 48 hours. Cells were then treated with either DMSO or 500 nM CPT for 2 hours and proteins were harvested. **A.** Western blot shows the impact of Rad18 down-regulation on monoubiquitination status of FANCD2 as indicated by (*asterisk), and levels of BRCA2 and Rad51 proteins. Densitometry analysis of blots from multiple experiments presented in histogram. **B.** Western blot showing the effects of FANCD2 knockdown on Rad18, Rad51, and BRCA2 protein levels basally and in response to CPT, densitometric analysis of blots from multiple experiments presented in histogram and the error bars indicate ± S.D.

As expected, exposure of H1299 cells to CPT elicited a robust DNA damage response by inducing Rad18, FANCD2, BRCA2 and Rad51 nuclear foci formation (Figure 1A and 1B). Cells treated with the vehicle (DMSO) alone exhibited little or no detectable foci of these proteins (data not shown). The FA pathway was activated in response to CPT as evidenced by the monoubiquitination of FANCD2 (Figure 2A) and its nuclear localization (Supplementary Figure S1C). Consistently, CPT exposure induced replication stress and associated DSB as indicated by γH2AX and FANCD2 foci and their co-localization with EdU (5-ethynyl-2′-deoxyuridine) (Supplementary Figure S1A) and induction of 53BP1 foci (Supplementary Figure S1B). As reported previously, Rad18 downregulation attenuated CPT-induced monubiquitination of FANCD2 (Figure 2A and Supplementary Figures S2A and S2B), its nuclear localization (Supplementary Figure S1C) and led to an approximately 50% decrease in its foci formation (Figure 1A and 1C). However, FANCD2 depletion did not cause a significant decrease in Rad18 nuclear foci formation (Figure 1A) in response to CPT. Moreover, either downregulation of Rad18 or FANCD2 in these cells, substantially decreased CPT-induced BRCA2 (≈50%), and Rad51 (≈50%) foci and their co-localization (Figure 1A, 1B, 1C and Supplementary Figure S4B and S4D). Similarly, knocking down either Rad18 or FANCD2 also resulted in decrease of basal and CPT-induced levels of BRCA2 and Rad51 proteins compared to their respective controls (Figure 2A and 2B). However, further studies are needed to fully elucidate the molecular basis for this decrease in BRCA2 and Rad51 protein level. To rule out that these effects are cell line specific or off-target effects of siRNAs, these results were further confirmed in A2780 (ovarian cancer) cells (Supplementary Figure S2A) and using an siRNA targeting 3′-UTR region of Rad18 transcript (Supplementary Figure S2B). Furthermore, consistent with previous studies [12, 29], E3 ubiquitin ligase activity of Rad18 is important for the FANCD2 foci formation (Supplementary Figure S3A), and its nuclear localization (Supplementary Figure S3B). Collectively, these results indicate a functional relationship between these four proteins in response to CPT-induced DNA damage. Recently, FANCD2 has been shown to form a functional complex with Rad18 and Rad51 and rescue stalled replication forks resulting from dNTP depletion due to HU treatment [45, 46]. FANCD2 also directly interacts with BRCA2, and this functional interaction is necessary for proper BRCA2 foci formation and efficient repair of DSB by HR [43, 47, 48]. Based on these observations, our data indicate that Rad18 may act upstream to other three proteins and regulate this functional interaction by promoting FANCD2 monoubiquitination and its nuclear localization in response CPT-induced fork-stalling lesions.

### BRCA2 and Rad51 act downstream of Rad18 and FANCD2 in response to CPT-induced DSB

**Figure 3:**
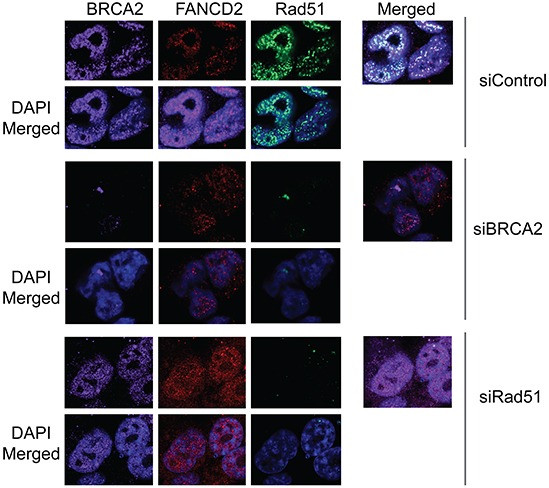
Downregulation of Rad51 or BRCA2 does not significantly effect on CPT-induced foci formation of Rad18 and FANCD2 H1299 cells were treated with siRNAs directed against either Rad51 or BRCA2 or control siRNAs and were exposed to 500 nM CPT for 2 hours, fixed and labeled with the indicated antibodies and stained with fluorophore-labeled secondary antibodies and visualized by immunofluorescence microscopy. Nuclei were stained with DAPI. Cells treated with DMSO did not demonstrate any significant, detectable foci and are not shown. (A) The formation of BRCA2, FANCD2 and Rad51 foci in response to CPT-induced DSB in cells depleted in either BRCA2 or Rad51 compared to control siRNA-treated cells. The extent of co-localization of the three proteins is shown in their respective right panels labeled as “merged”.

**Figure 4:**
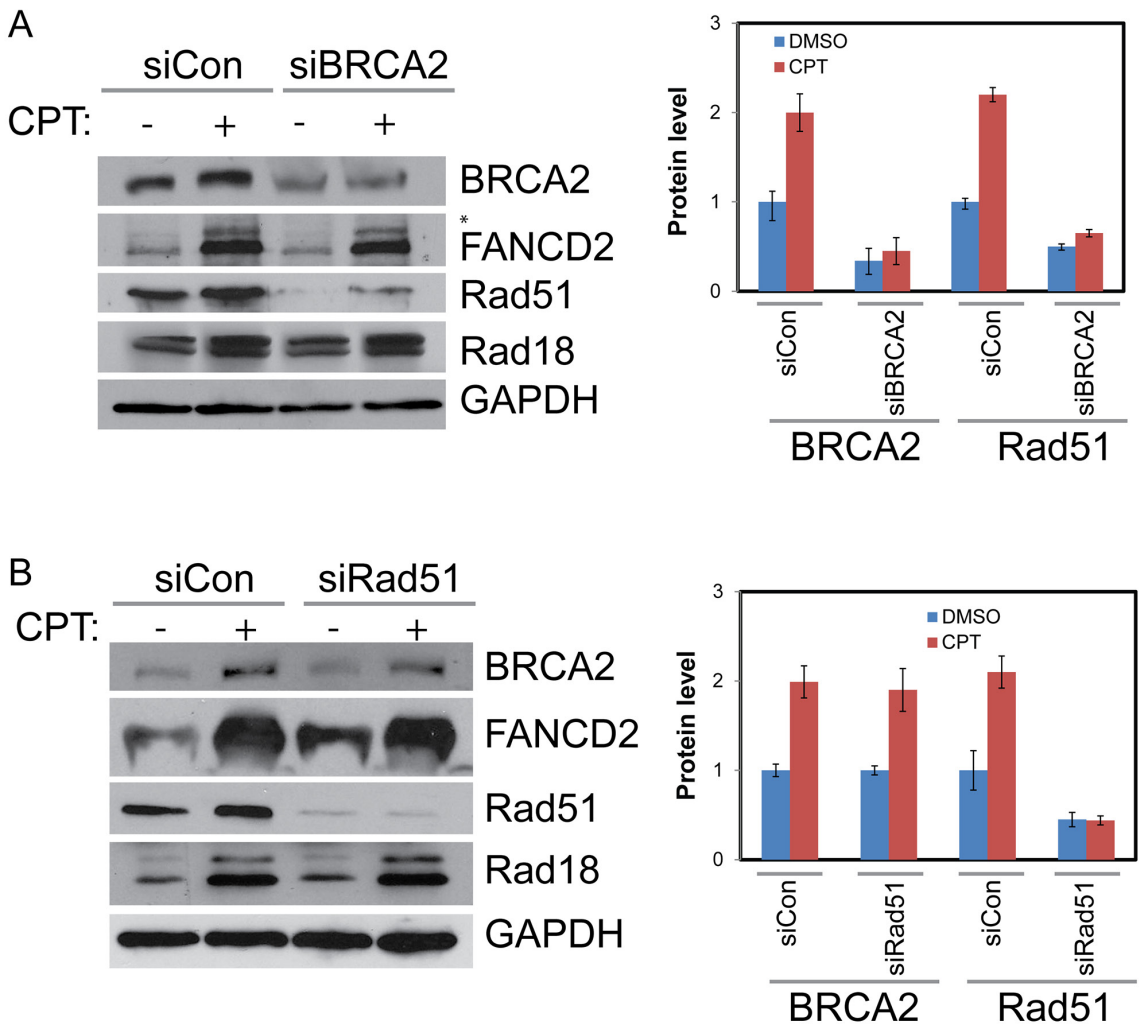
BRCA2 increases stability of Rad51 in CPT-treated cells but Rad51 has no detectable impact on BRCA2, Rad18 or Rad51 **A.** H1299 cells were depleted of BRCA2 by siRNA or treated with a control siRNA for 48 hours. They were then treated with vehicle or 500 nM CPT for 2 hours and proteins were harvested. Western blot shows downregulation of BRCA2 diminishes Rad51 protein levels basally and in response to CPT treatment but has little impact on Rad18 or FANCD2. **B.** Rad51 knockdown has no significant impact on BRCA2, Rad18 protein levels and monoubiquitination of FANCD2 (as indicated by *asterisk) basally and in response to CPT. Histogram in the right panels shows the densitometric analysis of blots from multiple experiments and the error bars indicates ± S.D. of.

To determine whether BRCA2 and Rad51 have any reciprocal effect on Rad18 and FANCD2 foci formation and stability, H1299 cells were transfected with BRCA2 and Rad51 siRNAs and their responses to CPT were monitored. Transient depletion of BRCA2 did not alter FANCD2 monoubiquitination (Figures 3, 4A and Supplementary Figure S4A) or Rad18 protein levels or their foci formation in response to CPT (Figure 4A, Supplementary Figure S4C and data not shown). However, BRCA2 knockdown resulted in decrease of Rad51 protein (Figure 4A) and its CPT-induced foci formation (Figure 3 and Supplementary Figure S4B). These findings suggest that BRCA2 may act downstream of both Rad18 and FANCD2 in CPT-induced DNA damage response.

**Figure 5:**
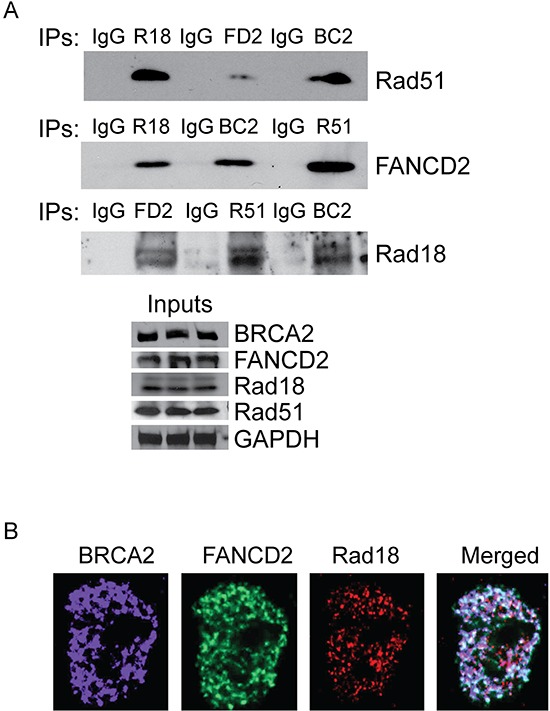
Rad18, FANCD2, BRCA2 and Rad51 co-immunoprecipitates and co-localizes in response to CPT H1299 cells were treated with 500 nM CPT for 2 hours and proteins were isolated and immunoprecipitated with antibodies against Rad51, FANCD2 BRCA2 and Rad18. Immunoglobulin G (IgG) used as negative control. **A.** Each immunoprecipitated sample was tested for the presence of the other 3 proteins by Western blots and the bottom panel shows the equal levels of proteins in input protein samples. **B.** Rad51 was knocked down in H1299 cells and treated with 500 nM CPT for 2 hours and the co-localization of the remaining three proteins was visualized by immunofluorescences. The images show that Rad51 is not required for the co-localization of the Rad18, FANCD2 and BRCA2 proteins in response to CPT.

Interestingly, Rad51 depletion had no significant impact on either the protein levels (Figure 4B) or the CPT-induced foci formation of Rad18, FANCD2, or BRCA2 (Figures 3, 4B, and Supplementary Figures S4A, S4C, S4D, and S5). Furthermore, Rad51 does not seem to alter co-localization of the other three proteins (Figure 3, merged panels and Figure 5B). Conversely, Rad51 protein levels and its foci formation were diminished by depletion of Rad18, FANCD2 and BRCA2 (Figures 1A, 1B, 2A, 2B, 3, 4A and Supplementary Figure S4B). These results suggests that Rad51 acts downstream of Rad18, FANCD2 and BRCA2 in response to CPT and its levels or stability depends on the status of the other three proteins.

### Rad18, FANCD2, BRCA2, and Rad51 interact and co-localize at the CPT-induced DSB

Rad18, FANCD2, BRCA2 and Rad51 all are known to be involved in DSB repair by HR. Several studies demonstrated the molecular interactions between some of these proteins and their interdependence [40–46]. Based on these observations, our data suggests that these proteins may act together in the repair of CPT-induced DNA lesions and may promote stability, activation, and/or ability to form DNA repair foci of each other. Therefore, it is important to determine if these proteins form a functional complex or interact, directly or indirectly, in response to DNA damage. To this end, co-immunoprecipitation assays were performed in cells treated with CPT. The protein levels were normalized for each immunoprecipitation reaction (Figure 5A, bottom panel) and immunoglobulin G (IgG) was used as a negative control. As expected, Rad18, FANCD2, BRCA2 and Rad51 all co-immunoprecipitated each other (Figure 5A, top panel), suggesting that these proteins all interact directly or indirectly to form a functional complex in response to CPT. To further confirm whether Rad18 is required for these interactions, the ability of FANCD2 to immunoprecipitate Rad51 and BRCA2 in cells treated with siRad18 was tested. When Rad18 was depleted, FANCD2 failed to immunoprecipitate BRCA2 and Rad51 proteins (Supplementary Figure S6), suggesting Rad18 is important for these functional interactions. These differences could be due to the decreased levels of the proteins or that Rad18 is necessary for the interactions between FANCD2, BRCA2 and Rad51. However, further work is needed to determine the molecular basis of these interactions and if all of these proteins are part of a large DNA repair complex. Interestingly, in these immunoprecipitations, a significant amount of the FANCD2 pulled down was the monoubiquitinated form in control cells (Supplementary Figure S6, indicated by asterisk), whereas in Rad18-deficient cells it is mostly unmodified FANCD2.

### Rad18, FANCD2, BRCA2 and Rad51 work in a common pathway to repair CPT-induced DSB by HR

**Figure 6:**
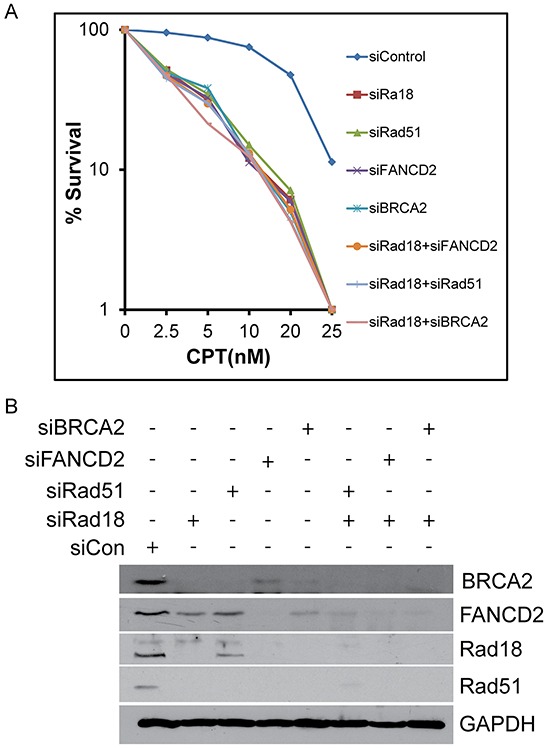
Depletion of Rad18, FANCD2, BRCA2 or Rad51 singly or in combination with Rad18 sensitizes cells to CPT to similar extent H1299 cells were treated with siRNA to knock-down Rad18, FANCD2, BRCA2 and Rad51 alone or in combination with Rad18. These cells were treated overnight with the indicated concentration of CPT or DMSO and surviving cells were allowed to form colonies. Colonies containing at least 25 cells were counted and plotted (top panel). The clonogenic survival assays were performed in triplicates and graph represents means of three independent experiments with error bars representing the ± S.D. The bottom panel shows a representative Western blot to confirm depletion of the targeted proteins.

Replication-coupled DSB, such as those induced by CPT, are predominantly repaired by HR-mediated repair [18]. Deficiencies in Rad18, FANCD2, BRCA2, and Rad51 are all known to sensitize tumor cells to CPT. Data shown above suggest that BRCA2, FANCD2, Rad18, and Rad51 functionally interact and may enhance the repair of DNA lesions induced by CPT by HR. To further examine the coordination of these proteins in the HR process, each gene was knocked down singly or in combination with Rad18 and assessed for sensitivity to CPT by clonogenic survival assay. Depletion of Rad18, FANCD2, BRCA2 and Rad51 all resulted in similar levels of CPT hypersensitivity compared to control cells (Figure 6A and 6B). Additionally, when BRCA2, FANCD2 and Rad51 were knocked down in combination with Rad18, there was little change from the single knockdown samples. To rule out that these results are specific to one cell line, these results were further confirmed using Rad18 wild-type and Rad18-null HCT116 cells transfected with siRNAs for FANCD2, Rad51 and BRCA2 (Supplementary Figure S7A and S7B). In these cells, there was no significant difference between the Rad18 KO cells and those transiently depleted for any of the other proteins. These results further indicate that Rad18, FANCD2, BRCA2, and Rad51 function together in the same pathway to repair CPT-induced, replication-coupled DSB.

**Figure 7:**
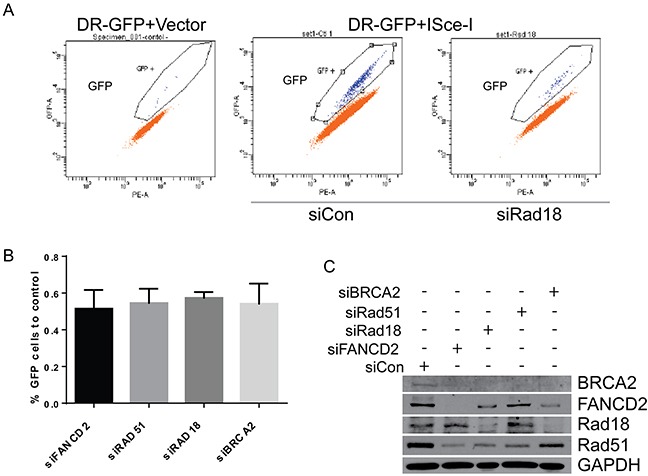
Downregulation of Rad18, FANCD2, BRCA2 and Rad51 results in similar levels of HR deficiency in ISce-I induced DSB repair H1299 cells stably expressing the DR-GFP reporter were transiently transfected with siRNAs directed against Rad18, FANCD2, BRCA2 and Rad51, or a control siRNAs in addition to an expression vector for the restriction enzyme I-SceI or an empty vector. I-SceI causes a site specific DSB in the DR-GFP cassette, and repair of this DSB by HR allows GFP expression which can be quantitated by flow cytometry to measure HR. **A.** Representative flow analyses for GFP in cells without the I-SceI restriction enzyme (1^st^ panel), with fully function HR (control siRNA; panel 2), and after depletion of Rad18 (panel 3). **B.** Combined data from at least three separate experiments plotted as a percentage of control siRNAs GFP production (i.e., percentage of normal HR efficiency). The bars represent S.E.M. and all are statistically significant to control (P<0.05). **C.** Western blot from one representative experiment showing level of siRNA knockdown for each protein.

To further assess the contributions of each of these proteins in repair of DSB by HR, DR-GFP reporter assays were performed utilizing site specific endonuclease I-SceI that generates a DSB in a GFP reporter. Background GFP expression is very low as determined by an I-SceI negative control (Figure 7A, first panel). Cells transfected with control siRNA exhibited GFP expression and represent the normal HR repair capability (Figure 7A, second panel). Depletion of Rad18, FANCD2, BRCA2 and Rad51 each exhibited similar decrease (all around 50% of control) in the HR efficiency in these cells (Figure 7B and 7C). Therefore, BRCA2, FANCD2, Rad18 and Rad51 each appear to contribute to repair and function in the HR pathway.

### Functional interaction between Rad18, FANCD2, BRCA2 and Rad51 suppresses error prone pathways in repair of CPT-induced DNA lesions

**Figure 8:**
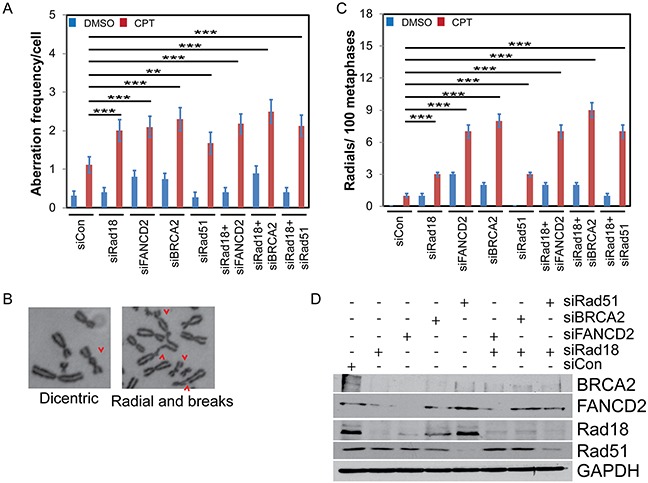
Knocking down Rad18, FANCD2, BRCA2 or Rad51 increases frequency of CPT-induced chromosomal aberrations (CA) and radials H1299 cells were treated with siRNA to knock-down BRCA2, FANCD2, Rad18 or Rad51 singly or in combination with Rad18 or control siRNA. Cells were treated with vehicle (DMSO) or 500 nM CPT and colcemid for 2 hours, fixed and harvested for chromosome isolation. The chromosomes were deposited on a slide, stained and analyzed for the presence of aberrations and radial formation. A minimum of 50 metaphase spreads were analyzed. **A.** The frequency of aberrant chromosomes formed during CPT treatment in cells depleted for the indicated protein(s) normalized per cell. **B.** Representative images indicating the range of CA found under these conditions. A wider variety and more examples and data from different knockdowns can be found in Supplemental Figure S6. **C.** The number of radial chromosomes found in CPT-treated cells depleted for the protein(s) indicated. Both graphs (A) and (C) show the means from three independent experiments. Error bars represent the standard error of the mean. **D.** Western blot from one representative experiment showing levels of knockdown achieved. ** indicates statistical significance at P< 0.05 and *** indicates P<0.001.

In S-phase, Rad18, FANCD2, BRCA2, and Rad51 were all found to play important roles in maintaining stability of stalled replication forks and timely repair of collapsed forks by error free HR to maintain genome integrity [45–48]. Thus, deficiency in these genes compromises HR efficiency, which could lead to cell death or repair through error prone recombination mechanisms, such as non-homologous end joining (NHEJ), increasing the frequency of chromosomal aberrations (CA). Deficiencies in FA-BRCA-Rad51 tumor suppressor group have been shown to induce gross CA in response to a variety of genotoxins [49–51]. To assess whether Rad18 deficiency also enhances CA frequency, we downregulated these genes in H1299 cells alone or in combination with Rad18. Metaphase spreads were scored for CA in cells treated with either vehicle (DMSO) or CPT. Figure 8A shows a significant increase in the frequency of spontaneous CA formation in FANCD2- and BRCA2-depleted cells compared to controls. Upon exposure to CPT, cells deficient in any of the single genes or combined with Rad18 knockdown showed statistically significant increases in the CA frequency compared to control cells. Furthermore, the magnitudes of CA frequency in each of the knockdowns were similar. Examples of the CA produced by CPT are shown in Figure 8B and Supplementary Figure S8. Deficiency in FA genes has been shown to induce radial formation upon exposure to genotoxins [51]. Interestingly, a significant increase in spontaneous and CPT-induced radial chromosomes was observed in Rad18-deficient cells (Figure 8C). Although a similar frequency of gross CA was observed in single gene knockdowns and co-depletions with Rad18, the frequency of radial chromosomes was significantly higher in FANCD2- or BRCA2-deficient cells. This could be due to independent functions of these proteins in either interactions with other DDR proteins or their role in suppression of error-prone DNA repair pathways such as NHEJ [52–56].

### Rad18 stabilizes replication forks in CPT-treated cells

**Figure 9:**
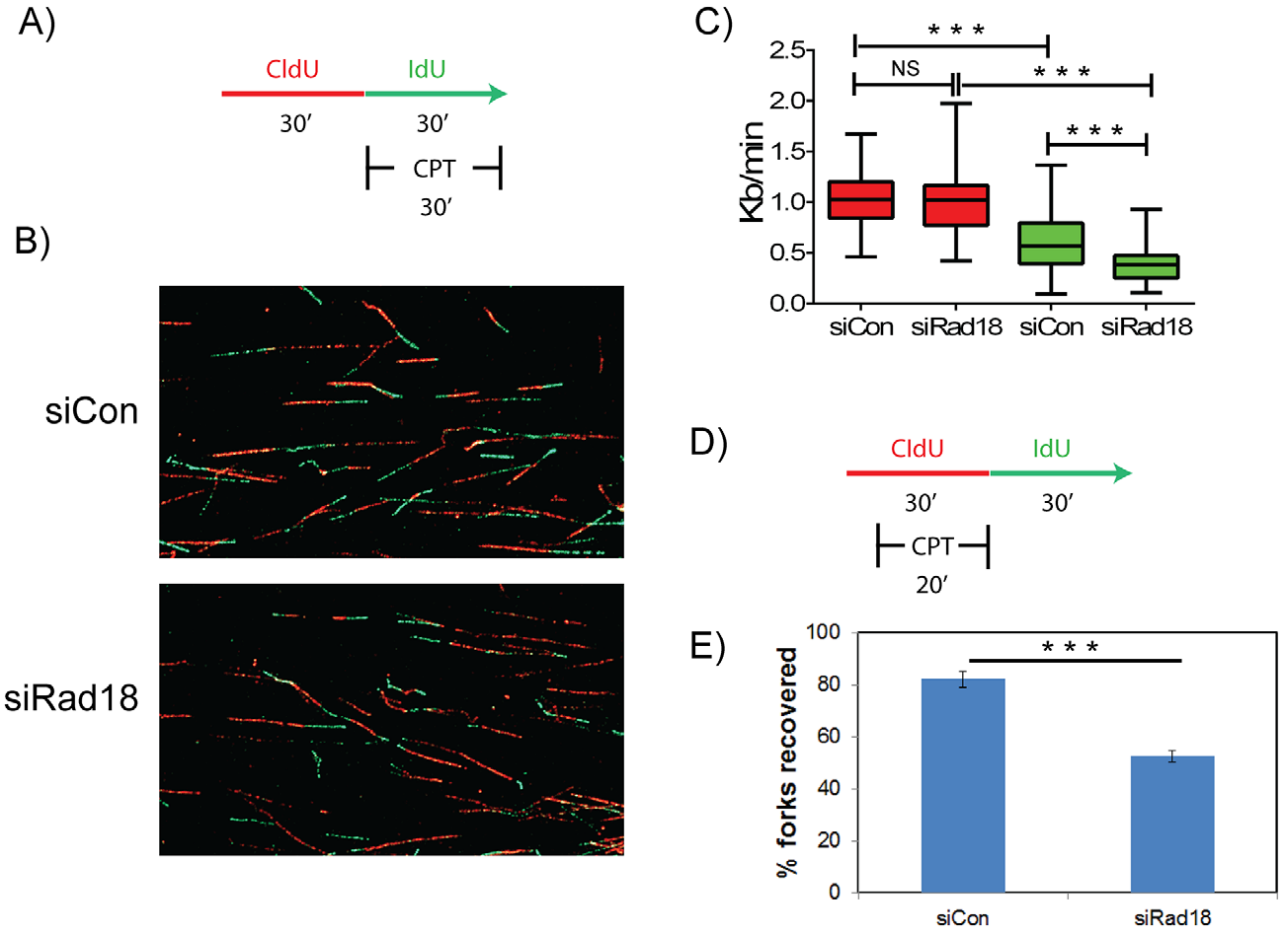
Rad18 is necessary for replication fork stability and recovery in response to CPT-induced replication blocks H1299 cells treated with siControl or siRad18 were pulsed with thymidine analogues CldU and IdU and analyzed for replication fork stability **A.** and recovery **D.** Representative images of DNA fibers in siControl and siRad18 cells **B.** The measurements of obtained DNA fibers did not show any significant difference in the fork velocity (P>0.05) between IdU-labelled and DMSO-treated siControl or siRad18 cells **C.** red bars). Whereas, upon treatment with CPT siRad18 cells showed a statistically significant (P<0.001) reduction in the fork velocity compared to siControl cells (C, green bars). Similarly, siRad18 cells treated with CPT also showed a statistically significant (P<0.001) decrease in the fork recovery compared to the siControl cells treated with CPT **E.** Statistically significant (P<0.001) groups are indicated with ***. NS indicates data not significant.

Several studies demonstrated important roles for FA-BRCA proteins in maintaining replication fork stability and restart of the stalled or collapsed forks in response to genotoxic stress [44]. To analyze the role of Rad18 in maintaining replication fork stability and recovery, DNA fiber assays were performed as described [44, 63, 64]. To measure the fork velocity in H1299 cells transfected with control or Rad18 siRNAs, cells were labeled with CldU for thirty minutes followed by labelling with IdU for thirty minutes in the presence or absence of CPT (Figure 9A). Transient depletion of Rad18 does not significantly alter fork velocity (siControl and siRad18 cells were 1.04 and 1.0 Kb/min, respectively). However, when cells were exposed to CPT, those cells deficient in Rad18 exhibited substantially decreased fork velocity (siControl: 0.59 and siRad18: 0.38 Kb/min) (Figure 9B–9C). Furthermore, to determine if Rad18 also affects the ability of replication forks to restart after CPT-induced fork stalling, cells were treated with CPT and labeled with nucleoside analogues as shown in Figure 9D. Interestingly, restart or reversal of stalled fork in the presence of CPT in Rad18-depleted cells was approximately half that of Rad18-proficient cells (Figure 9E). Taken together this data demonstrates that while Rad18 is not necessary for DNA replication to proceed under normal conditions, it is vital for maintaining the stability of replication forks when they encounter a block, such as CPT-generated Top1cc, likely through activation and recruitment of FA pathway and Rad51 and associated proteins.

## DISCUSSION

Camptothecin and its chemotherapeutic analogues cause replication-associated DNA strand breaks that are primarily repaired by homologous recombination [18, 20]. This could be due to the nature of the DNA lesions generated by the Top1 inhibitors, and/or the availability of homologous chromosomes in S and G2 phases. Indeed, repair of these breaks by NHEJ has been shown to be toxic to the cells [52]. Cells deficient in HR or associated proteins, such as some members of the FA and BRCA families, as well as Rad18, demonstrate hypersensitivity to CPT [12, 24, 25, 27]]. Rad51 is a firmly established member of the HR repair pathway, and BRCA2 has been shown to directly bind and stabilize Rad51 on single-stranded DNA to facilitate HR [43]. Interestingly, loss of either BRCA2 or FANCD2 was found to shift DSB repair away from HR and toward the more error prone NHEJ pathway. We and others have previously shown that E3 ubiquitin ligase Rad18 plays an important role in repair of fork-stalling lesions by promoting monoubiquitination of FANCD2 [12, 29, 30, 33, 34]. However, even though Rad18, Rad51, BRCA2, and FANCD2 are known to have roles in DSB repair, the interplay between these four proteins have not been thoroughly examined in the context of CPT-induced fork-stalling lesions. Here we show that Rad18 is important for the activation of FANCD2 (Figure 2A) and its nuclear foci formation (Figure 1A and 1C) and deficiency in either Rad18 or FANCD2 decreases the protein levels and foci formation of BRCA2 and Rad51 in response to CPT (Figures 1B, 1C, 2A, 2B and Supplementary Figures S4B and S4D). On the other hand, BRCA2 downregulation only affected Rad51 foci (Figure 3 and Supplementary Figure S4B) and protein levels (Figure 4A), whereas Rad51 was not necessary for either stability (Figure 4B) or the ability of the other three to form CPT-induced foci (Figure 3 and Supplementary Figure S4). Similarly, downregulation of BRCA2 and Rad51 did not significantly impacted on monoubiquitination of FANCD2 in response to CPT (Figure 4A and 4B). Collectively, these results indicate that Rad18 acts upstream of FANCD2 by promoting its monoubiquitination, and together these proteins regulate BRCA2 and Rad51 to promote efficient repair of CPT-induced lesions by HR. More studies need to be done to determine the mechanism through which Rad18 regulates the stability of the other proteins.

**Figure 10:**
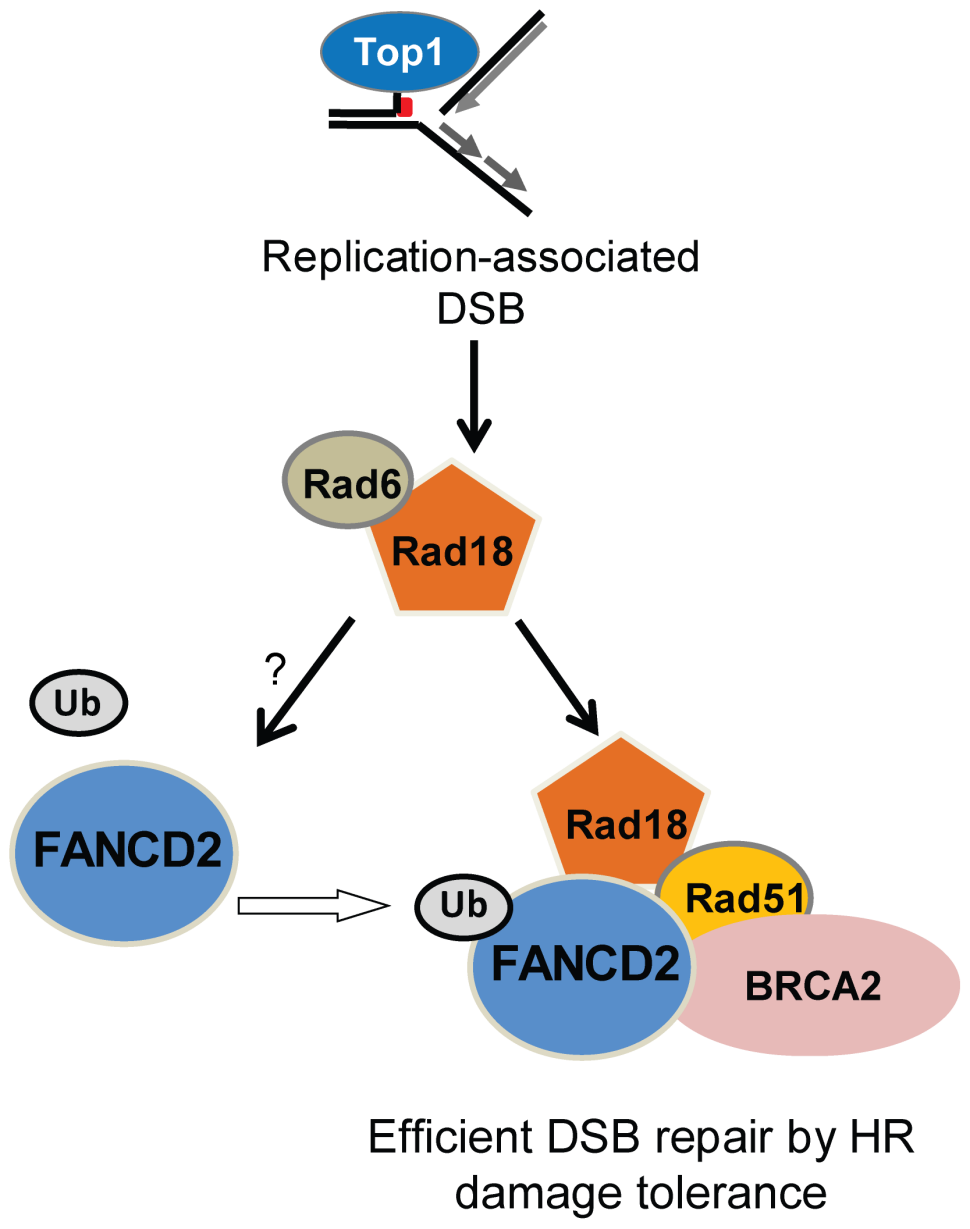
Summary of the data in a hypothetical model showing the functional relationships between Rad18, FANCD2, BRCA2 and Rad51 in repair of Top1-poisons-induced DSB In this model Rad18 acts first in this repair complex and promotes activation of FANCD2. This allows monoubiquitinated FANCD2, BRCA2 and Rad18 to form a repair complex with Rad51 and facilitates efficient repair of CPT-induced DSB by HR. Loss of Rad18 diminishes the formation of this repair complex and Rad51-mediated repair of DSB by HR, which may lead to repair of replication-associated DSB by alternative repair pathways such as NHEJ causing chromosomal aberrations and cell death.

The FA proteins (FANCs) are known to associate with replication forks and have been implicated to promote repair of stalled or collapsed forks by HR in association with BRCA proteins [36, 39]. Previously, Rad18 has been shown to directly bind Rad51C, a paralog of Rad51 (also known as FANCO) and localizes it to sites of DSB to promote HR [26]. In this study downregulation of Rad18, FANCD2, BRCA2 and Rad51 resulted in similar deficiencies in HR assays. Moreover, Rad18 and FANCD2 status dependent foci formation of BRCA2 and Rad51 in response to CPT suggests a functional interaction between these four proteins. Rad18 dependent co-immunoprecipitation and co-localization of all four proteins in response to CPT supports existence of such interactions (Figures 5A, 5B and Supplementary Figure S6). However, further studies needed to establish the mechanistic basis for these interactions. It is unknown if these four proteins are interacting in one complex or multiple groups of two or three proteins, or if the interaction is due to association through other protein(s). Consistent with this, knocking down any of these genes alone or in combination with Rad18 resulted in similar sensitivities to CPT and gross chromosomal aberrations and radial chromosome formation (Figure 8 and Supplementary Figure S8), indicating a switch to more error prone repair mechanisms such as NHEJ causing chromosomal aberrations and cell death [33]. Additionally, Rad18 deficiency caused decreased replication fork instability and fork recovery in the presence of CPT (Figure 9), suggesting an important role for Rad18 in faithful DNA replication. In summary, these findings suggest a model (Figure 10), where Rad18 promotes monoubiquitination of FANCD2, and associates with a functional complex involving Rad51, BRCA2, and FANCD2 in the repair of CPT-induced stalled or collapsed forks.

## MATERIALS AND METHODS

### Cell lines and reagents

H1299 (human non-small cell lung carcinoma) and A2780 (human ovarian cancer) were obtained from ATCC, and HCT116 and HCT116 Rad18^−/−^ (human colon cancer) cells were from Dr. Tadahiro Shiomi [27] and as used earlier [29]. Cells were cultured in Dulbecco's Modification of Eagle's Medium (DMEM) (Mediatech) supplemented with 10% fetal bovine serum (FBS) (Omega Scientific) and 1x Penicillin/Streptomycin sulfate (Gibco) [57]. Cells were routinely tested for mycoplasma contamination using Mycotest kit (Invitrogen) and were used prior to ten passages. CPT (Sigma) was used at concentrations and time periods indicated. Antibodies against the following targets were used: FANCD2, Rad51, 53BP1, GAPDH (all from Santa Cruz Biotechnology) BRCA2, RAD18 (Bethyl Laboratories) and γH2AX (Millipore). To express wild-type or the RING mutant Rad18, HCT116−/− cells were transfected with pcDNAmycRad18 and pcDNARad18 C28F, respectively.

### siRNAs and transfection

To knock down expression of each gene, the following siRNA sequences were used: control AGUUACUCAGCCAAGAACGAUU, FANCD2 GCACCGUAUUCAAGUACAAUU, Rad18 GAGCAUGGAUUAUCUAUUCAAUU and siRad18-3′UTR, 5′-UUA UAA AUG CCC AAG GAA AUU-3′ [12], Rad51 UGUAGCAUAUGCUCGAGCGUU [58] and BRCA2 AACUGAGCAAGCCUCAGUCAAUU [59]. All siRNAs were purchased from Dharmacon. Transfections of siRNA oligonucleotides were done using Lipofectamine 2000 (Invitrogen) following the manufacturer's protocol.

### Clonogenic survival assays

Cells were plated in triplicate in 6-well plates and treated overnight with indicated concentrations of CPT. Following drug treatment, cells were washed three times with PBS and three times with growth medium (CPT-free) to remove the drug, and allowed to form colonies. After 8 to 12 days, colonies were fixed in methanol and stained with crystal violet (0.5% w/v). Colonies containing more than 25 cells were counted either manually or using an automated imaging system (Gene Tools, Syngene), as described previously [60].

### Immunofluorescence

For immunofluorescence, cells were seeded in triplicate into glass bottom, 35 mm culture dishes (FluoroDish – World Precision Instruments) or 6-well plates. After approximately 48 hours, cells were treated with CPT or vehicle control (DMSO) as indicated. Cells were fixed at room temperature with 4% formaldehyde for 10 minutes and then in cold (−20°C) 100% methanol for 10 minutes. Fixed cells were then blocked in 10% goat serum for 30-60 minutes and washed 3 times with PBS. Cells were subsequently incubated overnight at 4°C with primary antibodies in PBS containing 5% BSA as described previously [57]. Cells were washed three or four times with PBS supplemented with 1% BSA then incubated with appropriate fluorophore-conjugated secondary antibody (IgG-Cy3, IgG-FITC or IgG-Cy5 – Invitrogen) for 2 h at room temperature. Detection of protein foci was done using the Nikon Ti eclipse confocal microscope and images were acquired at 100x and as described previously [57].

### Western blot analysis

Cells were grown in 10 cm cell culture dishes, transfected and treated as described above. Cells were washed in PBS and lysed in ice-cold cytoskeletal (CSK) buffer (10 mM PIPES (pH 6.8), 100 mM NaCl, 300 mM sucrose, 3 mM MgCl2, 1 mM EGTA, 1 mM dithiothreitol, 0.1 mM ATP, 1 mM Na3VO4, 10 mM NaF and 0.1% Triton X-100) freshly supplemented with protease and phosphatase inhibitors (Roche). Following determination of protein concentrations, gel samples were prepared in Laemmli buffer and heated to 100°C for fifteen minutes. Proteins were resolved by SDS-PAGE. Gels were electroblotted onto either a polyvinylidene fluoride (PVDF) or nitrocellulose membrane and blocked for 1 hour in 5% milk powder dissolved in Tris-buffered saline containing 0.1% Tween-20 (TBST). Membranes were incubated with primary antibody for 2 hours, followed by incubation with an appropriate horseradish peroxidase-conjugated secondary antibody (Sigma) in TBST for 1 hour. Bound antibody was visualized using a chemiluminescence detection kit from Millipore, following manufacturer's instructions and detected on film (CL-Xposure, Thermo Scientific) [61].

### Immunoprecipitation

Cells were grown and treated as detailed above, washed thrice in cold PBS and incubated with ice-cold RIPA buffer containing 20 mM Tris. HCl, pH 7.4, 150 mM NaCl, 1% NP-40, 1 mM PMSF, 20 mM NaF, 1 mM sodium vanadate and protease inhibitors for 30 min on ice. The cell lysates were centrifuged for 30 min at 30,000 g. These pre-cleared lysates were immunoprecipitated with specified primary antibody overnight, followed by incubation with protein A-Sepharose beads (Santa Cruz Biotechnology). Interacting proteins were eluted with 2x Laemmli buffer and analyzed by Western blot [57].

### Metaphase spread detection and analysis

Cells were grown and transfected with siRNA as described in previous sections. Cells were treated with vehicle (DMSO) or 500 nM CPT and 0.1 μg/ml colcemid for 2 hours. Then, cells were harvested and placed in hypotonic solution (0.075 M KCl) for 20 min and subsequently fixed in Carnoy's fixative (methanol: acetic acid, 3:1) and deposited on microscope slides. Metaphase spreads were counted after fluorescence plus-Giemsa staining as described [57]. At least 50 metaphase spreads were analyzed for CA and radial formation and their mean ± S.E. were calculated for each sample.

### Homologous recombination assay

This method uses GFP reporter assay to measure HR activity and is described elsewhere [62]. Plasmids were obtained from Addgene. Human H1299 lung cancer cells were stably transfected with pDRGFP and selected for puromycin resistance (10 μg/ml). These stably transfected cells were grown to 60% confluency and transfected with a plasmid expressing the restriction enzyme I-*Sce*I (pCBASce1). This restriction enzyme cuts the reporter plasmid and when repaired by HR GFP is expressed. GFP was measured by flow cytometry using a BD FACSCanto™ II (BD Biosciences).

### DNA fiber assay

DNA fiber labeling analysis was used to assess DNA replication fork stability and fork recovery as described [63, 64]. In brief, for fork velocity, siControl and siRad18 H1299 cells were labelled with IdU for 30 mins followed by CldU for 30 mins with DMSO or CPT. For fork recovery, siControl and siRad18 H1299 cells were labelled with IdU for 30 mins with or without CPT in the last 20 mins followed by CldU for 30 mins. Cells were then harvested and resuspended in ice cold PBS. Then 2 μl of the cell suspension was deposited over the slide and 10 μl of lysis buffer (0.5% SDS, 200 mM Tris-HCl ph 7.4, 50 mM EDTA) was added. Slides were tilted to 15° to stretch the DNA fibers, air dried, fixed in 3:1 methanol: acetic acid, denatured in 2.5M HCL and blocked with 5% BSA in PBS. Then slides were incubated with anti-CldU Cy3 and anti-IdU Alexa Fluor 488 for an hour. More than 200 replication structures were measured for fork velocity and fork recovery, statistical analysis were performed using Prism 6 (GraphPad Software).

### Statistical analysis

The Foci counting data and clonogenic survival data presented are averages of three independent experiments. Error bars represent the mean ± S.D. All the data were analyzed either by using GraphPad Prism 6 or Excel 2010. The data presented in the manuscript are representative of three independent experiments unless otherwise mentioned.

## SUPPLEMENTAL FIGURES


