# Supplementary material for: Rad18 is required for functional interactions between FANCD2, BRCA2, and Rad51 to repair DNA topoisomerase 1-poisons induced lesions and promote fork recovery
Source: Oncotarget. 2016 Feb 8;7(11):12537–53. doi: 10.18632/oncotarget.7247 (PMC4914303; doi:10.18632/oncotarget.7247)
Supplement: Supplementary file 1 [file oncotarget-07-12537-s001.pdf]

SUPPLEMENTARY FIGURES

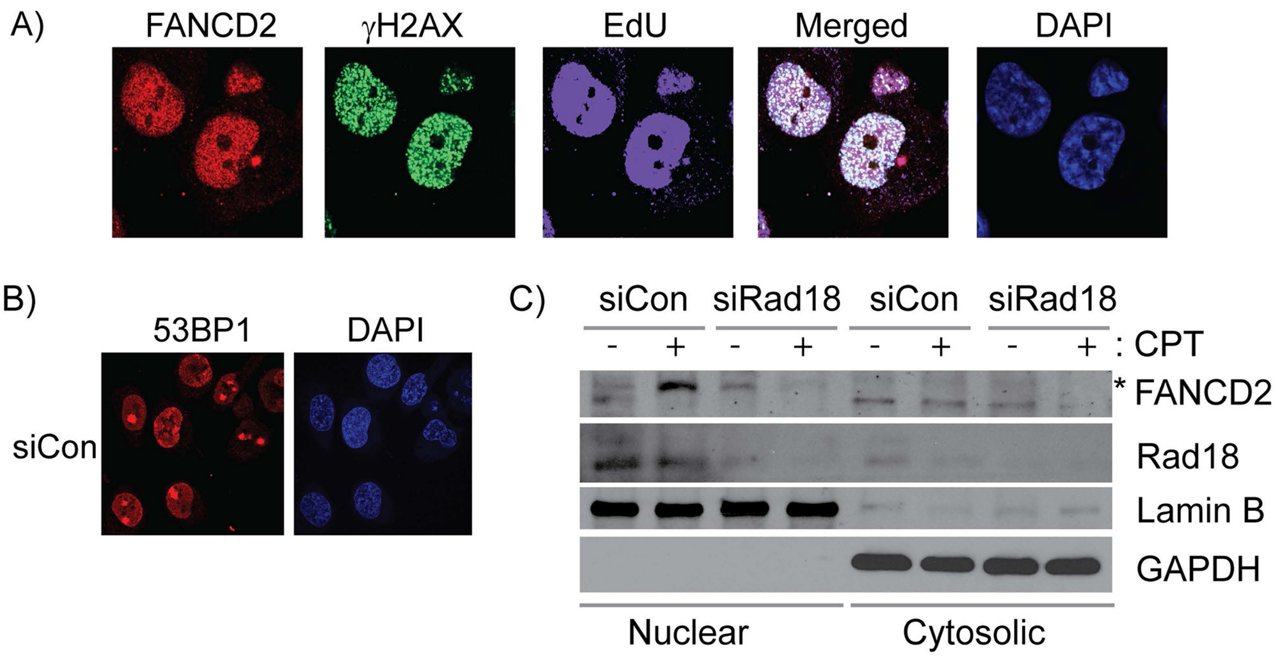

Supplementary Figure S1: Top1 poison CPT activates FA pathway and (as indicated by FANCD2 foci in red), induces replication associated DSB (as indicated by γH2AX and 53BP1). A. H1299 cells exposed to 500 nM CPT for 2 hours and before 3o min of fixation cells were labeled with Edu. CPT exposure induced FANCD2 (red), γH2AX (green) foci and they co-localized with regions of active replication stained with EdU (purple). **B.** To further confirm cell were also stained with 53BP1 another marker of DSB. **C.** Depletion of Rad18 blocks activation of FANCD2 and its accumulation in the nuclear fraction in response to CPT treatment. The asterisk (the FANCD band shift higher molecular weight) indicates monoubiquitinated form of FANCD2 present only in the nuclear fractions. The Lamin B and GAPDH blots for loading controls for the nuclear and cytosolic fractions respectively.

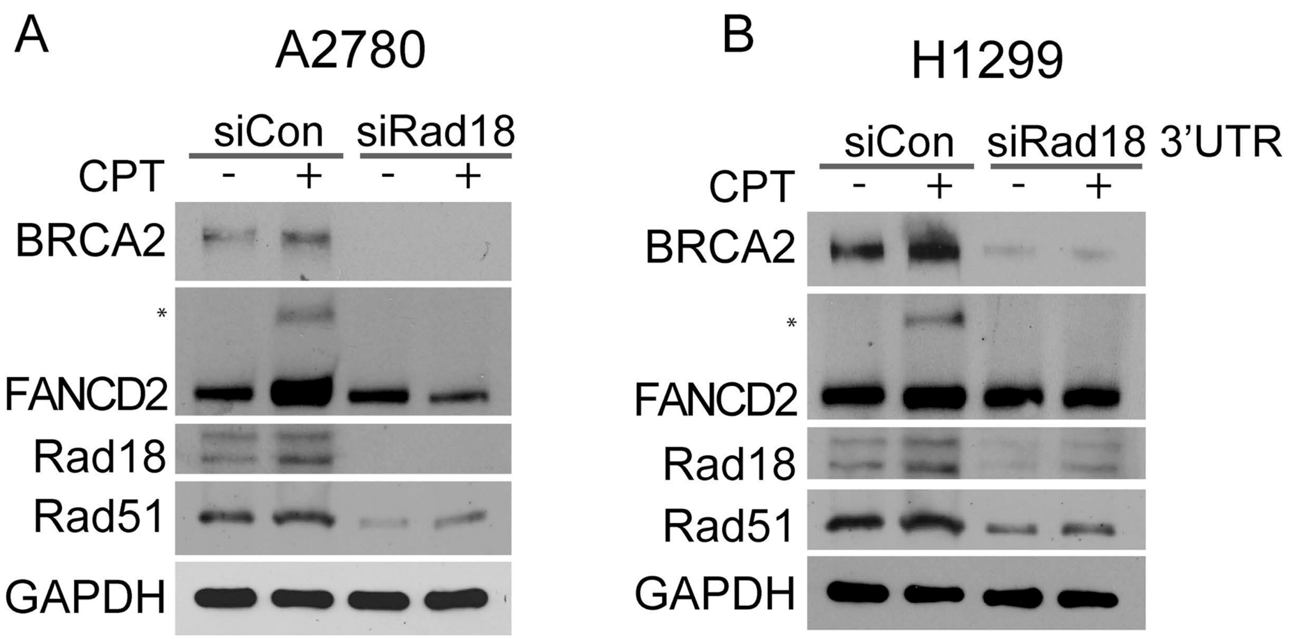

Supplementary Figure S2: Rad18-mediated activation of FA pathway (monoubiquitination of FANCD2) and decreased levels of BRCA2 and Rad51 proteins were not specific to one cell line (H1299) or off-target effects of siRNA. **A.** A2780 ovarian cancer cells were transfected with control siRNAs or targeting Rad18 were exposed to CPT and assessed FANCD2 monoubiquitination and BRCA2 and Rad51 protein levels. **B.** An siRNA directed against the 3′UTR of Rad18 was utilized to confirm the effects in this study were not off target effects of the siRNAs used.

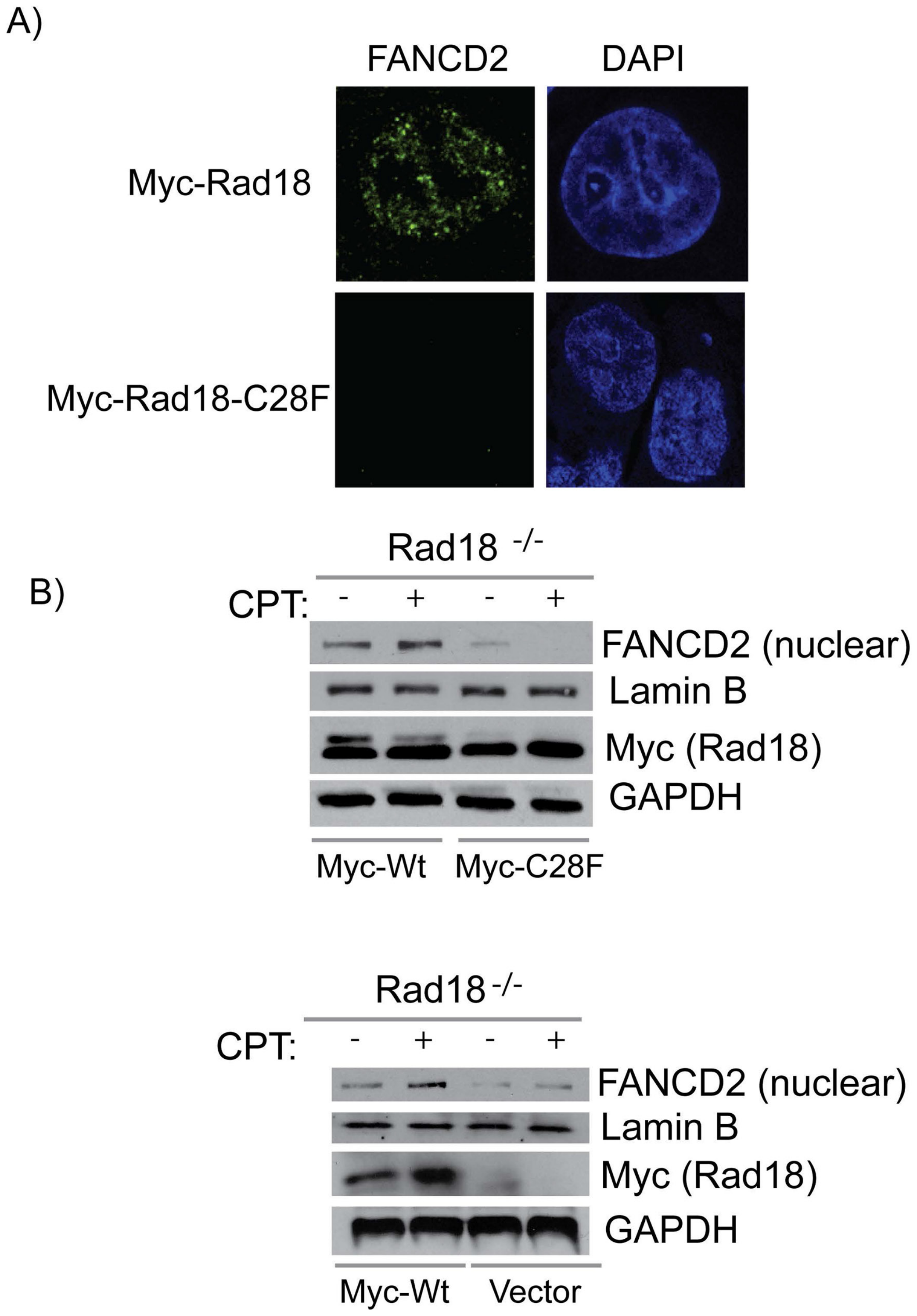

Supplementary Figure S3: Ubiquitin ligase function of Rad18 is required for activation of FANCD2. HCT116 Rad18$^{-/-}$ cells were transfected with plasmids expressing either wild-type or the RING mutant (C28F) of Rad18 or empty vector and exposed to CPT. **A.** Cells expressing the mutant Rad18 defective in ubiquitin ligase were not able to form FANCD2 foci in response to CPT. **B.** From the above cells fractionated protein lysates were used for Western blots, showing that RING mutant of Rad18 (upper panel) or empty vector (bottom panel) failed to complement CPT-induced monoubiquitination of FANCD2. CPT-induced increase in the monoubiquitinated FANCD2 (that is mainly present in the nuclear fractions, also see Supplementary figure 1C) requires wild-type Rad18.

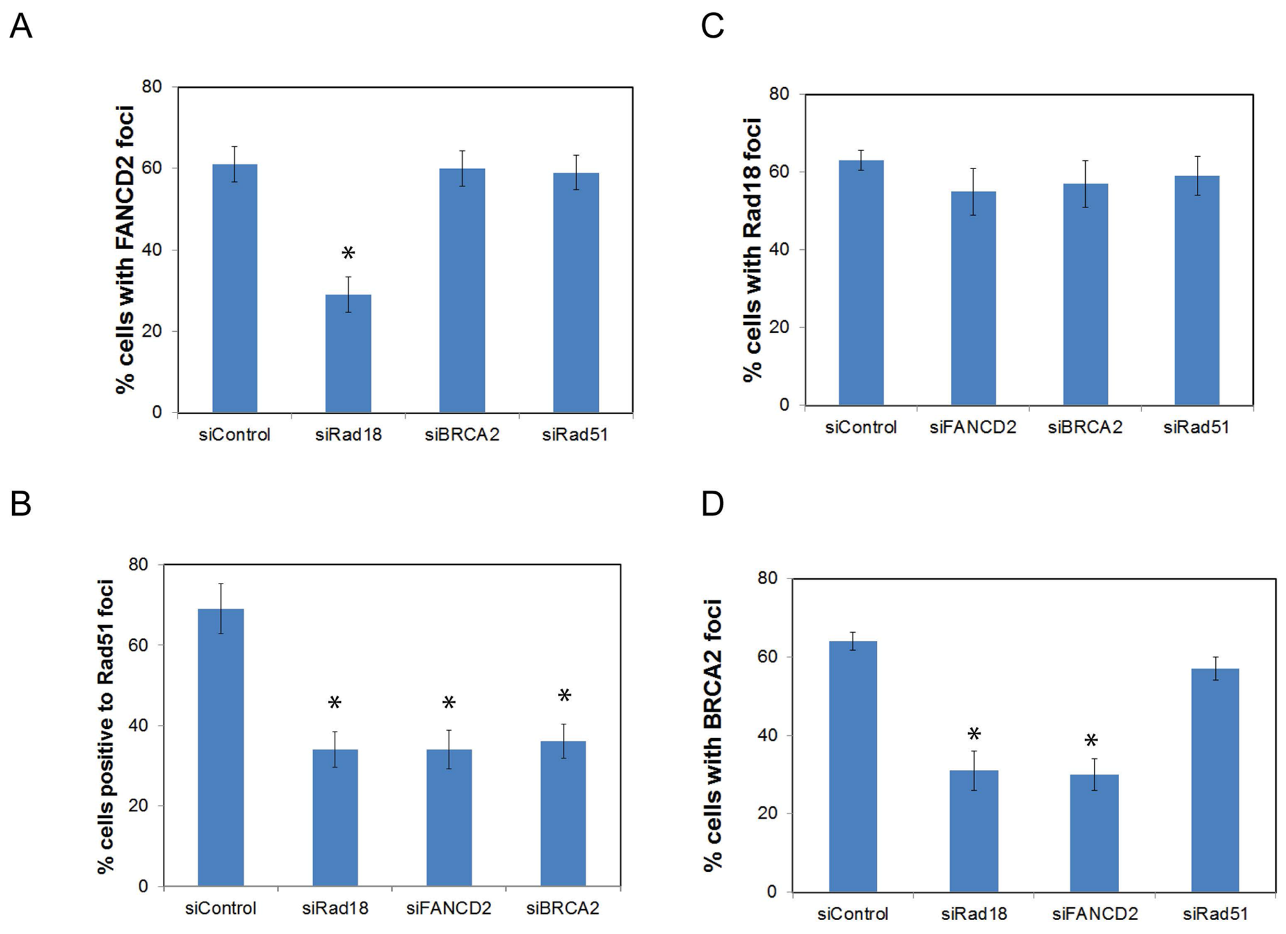

Supplementary Figure S4: Rad18 and FANCD2 required for the efficient foci formation of BRCA2, and Rad51 in response to CPT. H1299 cells were transfected with the indicated siRNAs and after 48 hours were exposed to 500 nM CPT for 2 hours, fixed and labeled with indicated antibodies and stained with fluorophore-labeled secondary antibodies. Cells with at least 5 foci were graded as positive and at least 15 random focal images (each containing 8-15 cells) were scored for each. The number of cells positive for **A.** FANCD2, **B.** Rad18, **C.** Rad51 and **D.** BRCA2 are presented. Each graph represents the mean of three independent experiment with error bars representing S.D. * Denotes statistical significance at $P<0.05$.

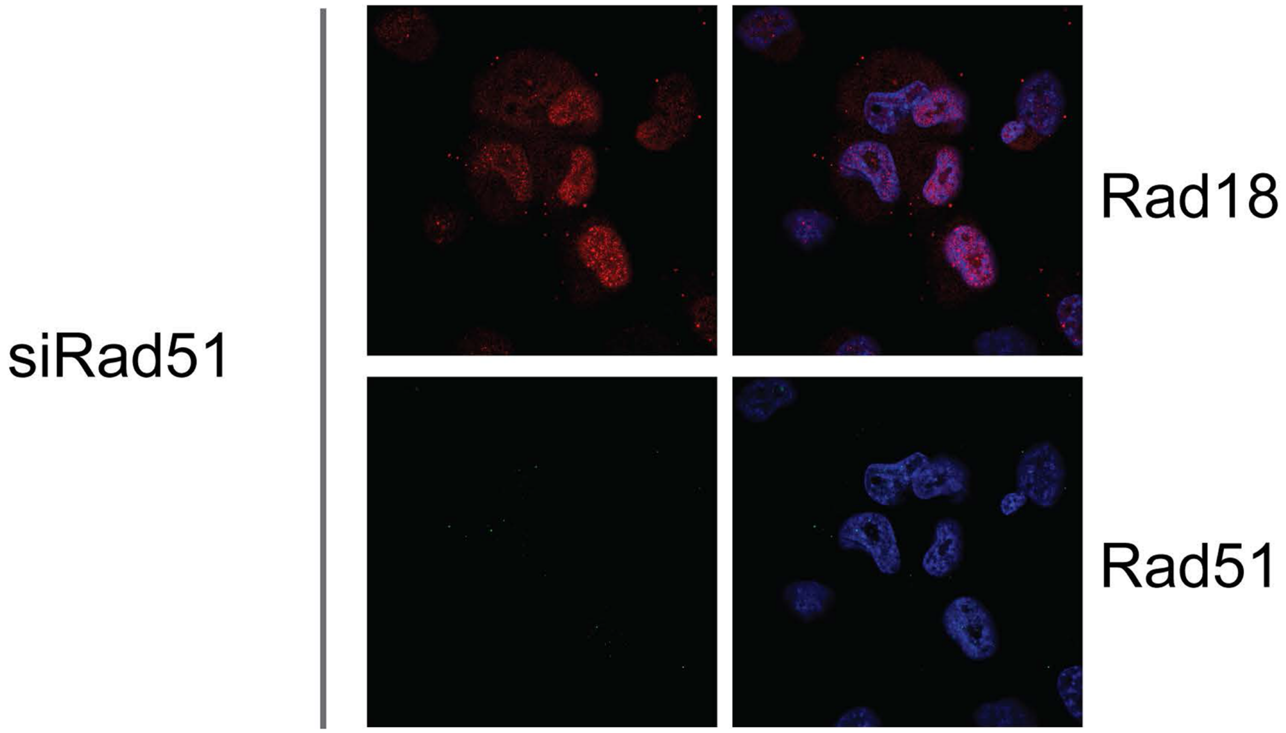

Supplementary Figure S5: Rad51 does not impact Rad18 foci formation in response to CPT. H1299 cells were depleted for Rad51, treated for 2 hours with 500 nM CPT, targeted with an antibody against either Rad18 or Rad51 and these proteins were subsequently visualized by treatment with a fluorophore-labeled secondary antibody.

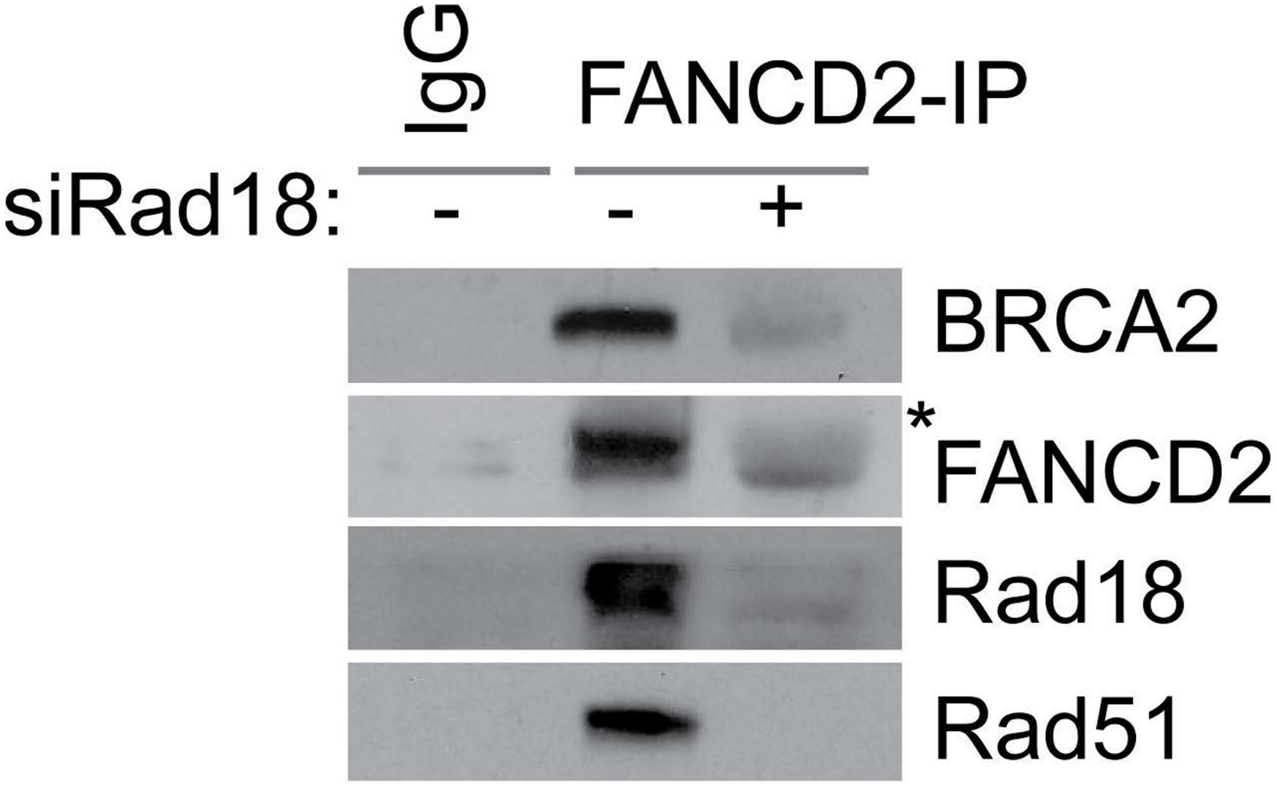

Supplementary Figure S6: Depletion of Rad18 disrupts FANCD2 interactions with BRCA2 and Rad51. H1299 cells were transfected with control or siRNAs or against Rad18 and exposed to CPT. Normalized protein lysates were used for immunoprecipitation with FANCD2 antibodies and IgG was used as a negative control to confirm interaction were antibody specific. The asterisk (*) indicates monoubiquitinated form of FANCD2, increased levels of this form present in the Immunoprecipitation of control siRNAs transfected cells and very little in the Rad18 downregulated cells. This figure is a companion to Figure 5.

A)

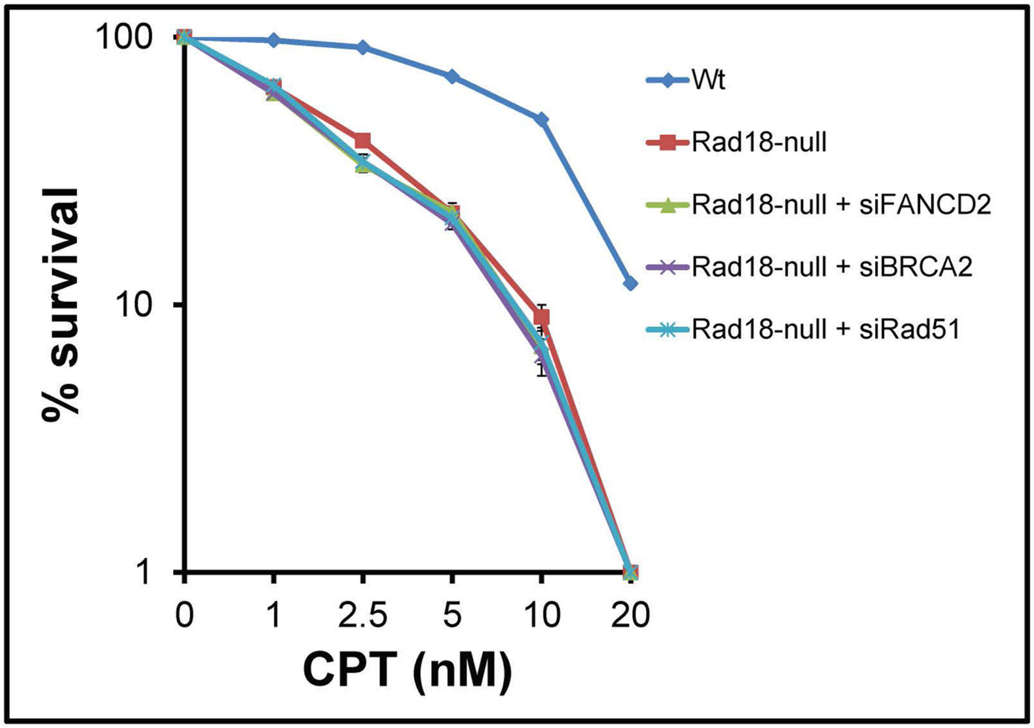

B)

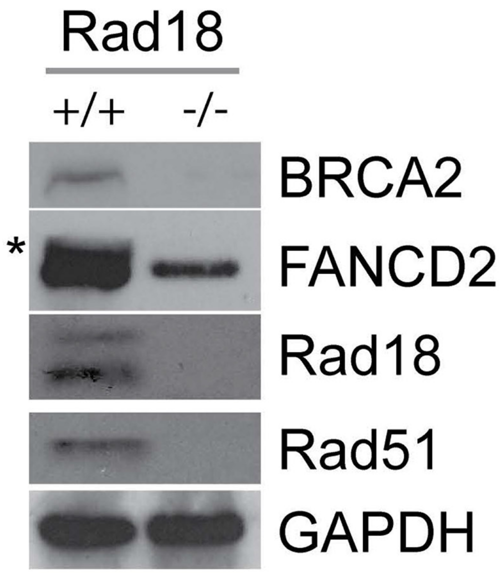

Supplementary Figure S7: Downregulation of FANCD2, BRCA2 and Rad51 in Rad18 null cells does not enhance sensitization to CPT. HCT116 Rad18 null cells were transfected with control, FANCD2, BRCA2 and Rad51 siRNAs and compared with HCT116 wild-type cells in clonogenic survival assays to assess their sensitivities to CPT. **A.** HCT116 cells deficient in Rad18 alone or in combination of FANCD2, BRCA2 and Rad51 exhibit similar sensitivity to CPT. **B.** The Rad18 null HCT116 cells have diminished levels of BRCA2, FANCD2, and Rad51 and the monoubiquitinated form of FANCD2, as indicated by asterisk (*) . This data is a companion to Figure 6.

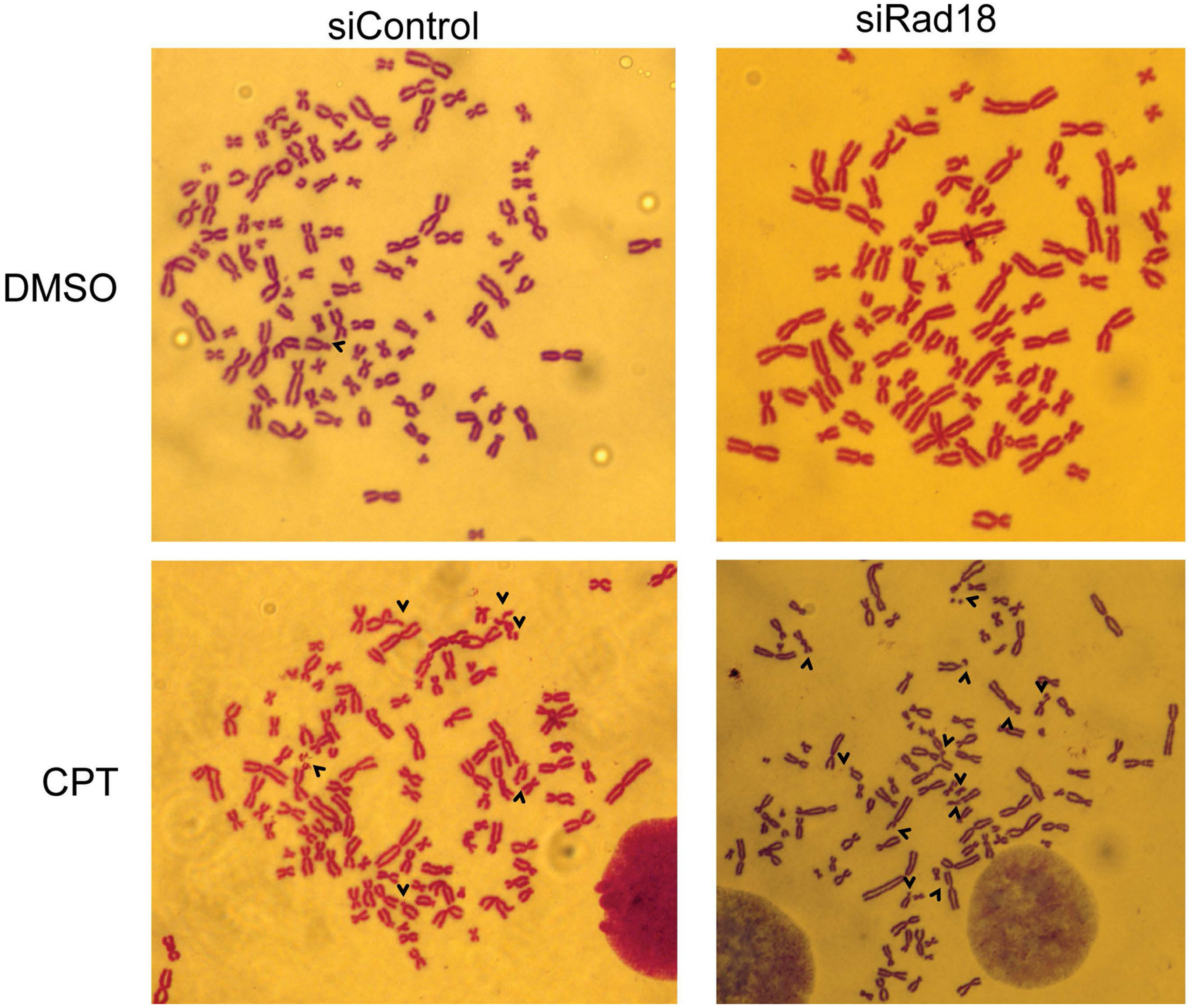

Supplementary Figure S8: Knocking down Rad18 increases frequency of CPT-induced chromosomal aberration and radials. This figure is a companion to Figure 8, showing more incidences of CA and radials in H1299 cells depleted of Rad18.
